# Validation of reference genes for expression analysis in the salivary gland and the intestine of *Rhodnius prolixus *(Hemiptera, Reduviidae) under different experimental conditions by quantitative real-time PCR

**DOI:** 10.1186/1756-0500-5-128

**Published:** 2012-03-06

**Authors:** Rafaela M Paim, Marcos H Pereira, Raffaello Di Ponzio, Juliana O Rodrigues, Alessandra A Guarneri, Nelder F Gontijo, Ricardo N Araújo

**Affiliations:** 1 Departamento de Parasitologia, Instituto de Ciências Biológicas, Universidade Federal de Minas Gerais. Bloco I4, Sala 177, Av. Antonio Carlos 6627, Pampulha, CEP 30270-901 Belo Horizonte, MG, Brazil; 2 Centro de Pesquisa René Rachou, Avenida Augusto de Lima, 1715, CEP 30190-002, Belo Horizonte, MG, Brazil; 3 Instituto Nacional de Ciência e Tecnologia em Entomologia Molecular, Rio de Janeiro, Brazil

## Abstract

**Background:**

*Rhodnius prolixus *is a blood-feeding insect that can transmit *Trypanosoma cruzi *and *Trypanosoma rangeli *to vertebrate hosts. Recently, genomic resources for invertebrate vectors of human pathogens have increased significantly, and *R. prolixus *has been one of the main species studied among the triatomines. However, the paucity of information on many of the fundamental molecular aspects of this species limits the use of the available genomic information. The present study aimed to facilitate gene expression studies by identifying the most suitable reference genes for the normalization of mRNA expression data from qPCR.

**Results:**

The expression stability of five candidate reference genes (*18S *rRNA, *GAPDH*, β-actin, α-tubulin and ribosomal protein *L26*) was evaluated by qPCR in two tissues (salivary gland and intestine) and under different physiological conditions: before and after blood feeding and after infection with *T. cruzi *or *T. rangeli*. The results were analyzed with three software programs: geNorm, NormFinder and BestKeeper. All of the evaluated candidate genes proved to be acceptable as reference genes, but some were found to be more appropriate depending on the experimental conditions. *18S*, *GAPDH *and α-tubulin showed acceptable stability for studies in all of the tissues and experimental conditions evaluated. β-actin, one of the most widely used reference genes, was confirmed to be one of the most suitable reference genes in studies with salivary glands, but it had the lowest expression stability in the intestine after insect blood feeding. *L26 *was identified as the poorest reference gene in the studies performed.

**Conclusions:**

The expression stability of the genes varies in different tissue samples and under different experimental conditions. The results provided by three statistical packages emphasize the suitability of all five of the tested reference genes in both the crop and the salivary glands with a few exceptions. The results emphasise the importance of validating reference genes for qRT-PCR analysis in *R. prolixus *studies.

## Background

Triatomines (Hemiptera, Reduviidae) are hematophagous insects and the vectors of *Trypanosoma cruzi*, the causative agent of Chagas disease in the Americas. For successful transmission, the parasite undergoes different stages of transformation in the gut of the insect vector until it is eliminated with the feces and urine [1]. Bugs from the genus *Rhodnius *can also be infected and transmit the protozoan *Trypanosoma rangeli*, which despite being non-pathogenic to humans and animals, can cause physiological damage to the insect vector [2,3]. Unlike *T. cruzi*, which develops exclusively in the gut of its invertebrate hosts, *T. rangeli *initially develops in the gut and then invades the hemolymph of the insect vector. The protozoan is transmitted to the vertebrate host through salivary secretion during feeding [2,4].

All nymphal and adult stages of triatomines feed exclusively on blood. The salivary glands and the intestine are the major organs involved in the interaction of the triatomine with trypanosomatids, and its vertebrate hosts and play a critical role in parasite development and blood intake during hematophagy [5-8].

In the last few years, the genomic resources for the invertebrate vectors of human pathogens have increased significantly [9]. Among the invertebrate vectors, the triatomine bug *Rhodnius prolixus*, the main vector of *T. cruzi *in the northern region of South America [10], has been studied. Sequences from *Rhodnius prolixus *are available to the scientific community, including more than 28,000 nucleotide sequences from transcriptomes and other studies [11,12] and more than 5 million contigs from the *Rhodnius prolixus *genome project (http://genome.wustl.edu/genomes/view/rhodnius_prolixus/). With the completion of the genome project, it is expected that the number of studies involving gene expression will increase.

Understanding the patterns of gene expression is important to provide insights into complex regulatory networks and will lead to the identification of genes relevant to new biological processes [13]. However, gene expression studies need robust normalization methods, which are necessary for the correction of non-specific variations, such as different amounts of starting material, inaccurate quantification of RNA, the quality of the RNA, and differences during cDNA synthesis that can trigger variations in PCR reactions. The most common method for normalizing gene expression levels is to normalize the mRNA levels of the gene of interest to endogenous control genes, often referred to as housekeeping or reference genes. Ideally, the housekeeping gene should not be regulated or influenced by the experimental procedure or co-regulated with the target gene. The housekeeping gene should also be expressed in abundance and have minimal innate variability [14].

Studies with triatomines and other insect models have shown that the expression levels of commonly used reference genes can differ among different tissue/organ types or physiological conditions [15-18]. In the present study, the expression level and stability of five potential reference genes were compared in two different tissues, the salivary glands and the crop (anterior midgut), from *Rhodnius prolixus *nymphs under two different physiological conditions: after blood feeding and after infection with trypanosomatids (*T. rangeli *and *T. cruzi)*. The results from this study will help in choosing suitable genes for the normalization of mRNA under conditions that are the focus of most studies on triatomines, such as before and after hematophagy and during the triatomine-trypanosomatid interaction.

## Methods

### Insects and parasites

*R. prolixus *were reared under controlled conditions of temperature (28 ± 2°C) and humidity (65 ± 5.0%) in a 12/12 h light/dark cycle and allowed to feed weekly on chickens or rats. Cultured epimastigotes from *T. rangeli *(CHOACHI strain) and *T. cruzi *(CL strain) were used. The epimastigote forms were cultured at 27°C in liver-infusion tryptose (LIT) medium supplemented with 15% fetal bovine serum, 100 mg/ml streptomycin and 100 units/ml penicillin. To maintain the infectivity, a cyclic infection of triatomines and mice with each parasite was performed every three months. Infection and bloodfeeding experiments followed the UFMG and FIOCRUZ guidelines on animal experimentation and were approved by the Ethical Committee on Animal Use (CEUA- FIOCRUZ-MG) under the number L-058/08.

### Blood feeding on rats

The salivary glands and the crop of third instar nymphs of *R. prolixus *were dissected at different starving times after molt (1, 3, 15 and 25 days) or different times after blood feeding (1, 3 and 7 days) on anesthetized rats. Samples were immediately frozen at -80°C until RNA extraction.

### Infection with *T. rangeli *and *T. cruzi*

For *T. rangeli *salivary gland infection, fourth instar nymphs were infected intracoelomically with 1 μl of PBS containing 100 parasites, and a control group was inoculated with PBS alone. The epimastigotes were obtained from 10-day-old culture medium, washed and resuspended in sterile PBS (0.15 M NaCl in 0.01 M sodium phosphate, pH 7.4). After 20 days of infection, the salivary glands of these insects were extracted and used in the experiments. Samples were immediately frozen at -80°C until RNA extraction.

For intestinal infection with *T. rangeli *or *T. cruzi*, the epimastigotes were obtained as above but were added to heat-inactivated rabbit blood at a concentration of 10^5 ^flagellates/ml. Second instar nymphs were allowed to feed on this blood through a membrane feeder (control insects were fed on non-infected blood). Fifteen days after the molt to the third instar, the insects in the chronic phase of infection had their crop extracted for experiments. Samples were immediately frozen at -80°C until RNA extraction.

### Selection of gene sequences and primer design

Five genes were selected for analysis: β-actin (*ACT*, GenBank ID: EU233794), α-tubulin (*TUB*, GenBank ID: ACPB02030650), the 60S subunit of a ribosomal protein L16 (*L26*, GenBank ID: ACPB02039040), glyceraldehyde-3-phosphate dehydrogenase (*GAPDH*, GenBank ID: ACPB02038754) and *18S *ribosomal RNA (*18S*, GenBank ID: AJ421962) (Table 1).

**Table 1:** Description, primer sequence and amplicon characteristics for the five candidate reference genes tested

Symbol	Name	Function	Primer Sequences	Amplicon size (bp)	E (%) *	R^2 ^**
*ACT*	β-actin	Involved in cell motility, structure and integrity	*For 5' *AATCAAGATCATTGCTCCACCAG *3' Rev 5' *TTAGAAGCATTTGCGGTGGAC *3'*	151	92%	0.996
*GAPDH*	Glyceralde hyde-3- phosphate dehydrogen ase	Glycolysis	*For 5' *GATGGCGCCCAGTACATAGT *3'* *Rev 5' *AGCTGACGGGGCTGTTATTA *3'*	111	96%	0.9906
*TUB*	α-tubulin	Cytoskeleton structural protein	*For 5' *TTTCCTCGATCACTGCTTCC *3'* *Rev 5' *CGGAAATAACTGGGGCATAA *3'*	129	90.1%	0.9978
*L26*	60S ribosomal protein	Structural constituent of ribosome	*For 5' *AGGTGGACAAAGATCGCAAG *3'* *Rev 5' *AAGTGTCCATTGCTGTCGTG *3'*	117	93%	0.9991
*18S*	18S ribosomal RNA	Cytosolic small ribosomal subunit	*For 5' *TCCTTCGTGCTAGGAATTGG *3'* *Rev 5' *GTACAAAGGGCAGGGACGTA *3'*	105	100%	0.9617

*Real-time qPCR efficiency (calculated by the standard curve method)

** Regression coefficient calculated from the regression line of the standard curve

The sequences were obtained from the contigs from the genome project (http://genome.wustl.edu/pub/organism/Invertebrates/Rhodnius_prolixus/assembly/Rhodnius_proli xus-3.0.1/output/). The primers used in the PCR reactions were designed with Primer3 software (http://primer3.sourceforge.net) using a maximum amplified fragment of 150 bp and a melting temperature (Tm) of approximately 60°C (Table 1).

### Total RNA isolation, cDNA synthesis and PCR

Total RNA was extracted from three pools of 10 crops or three pools of 20 salivary glands from each group with the Nucleospin^® ^RNA XS (Macherey-Nagel) according to the manufacturer's instructions. DNAse treatment was performed on column according to the Nucleospin^® ^RNA XS (Macherey-Nagel) manufacturer's instructions. The RNA was quantified using a NanoDrop^® ^ND-1000 (Thermo Scientific) at 260 nm. Only intact RNA was used for the reactions. RNA should have the ratio 260/280 (absorbance at 260/absorbance at 280) between 1.8 and 2.0, and the subunits of 28S and 18S clearly observed by native agarose gel electrophoresis. RNA (0.5 μg) was used for cDNA synthesis with 0.5 μg of random hexamers (Promega) using the M-MLV reverse transcriptase system (Promega) in a final volume of 25 μl. The product was used as a template for PCR reactions of housekeeping genes, which were performed for 35 cycles (94°C for 30 s, 60°C for 30 s and 72°C for 45 s) with 1 μl of cDNA, 200 nM of each primer, 200 μM of dNTPs and 1 U of Taq polymerase (Phoneutria) in a final volume of 20 μl.

### Cloning and sequencing of DNA

The PCR products were cloned into the pGEM-T Easy vector (Promega). After transformation into DH5α competent cells, recombinant plasmid DNA was isolated using the Wizard Plus SV Miniprep kit (Promega). The DNA inserts of the recombinant clones were amplified by PCR with universal primers - an M13 forward primer and an M13 reverse primer - derived from the vector. The sequencing reactions were conducted using the dideoxy method [19], and the samples were run on a MegaBACE 1000TM sequencing system (Amersham Biosciences). Each clone was sequenced twice in both directions, and the consensus sequence was derived from these sequences using the programs Phred v.0.20425 [20,21], Phrap v.0.990319 (http://www.phrap.org/), and Consed 12.0 [22]. The nucleotide sequences were compared with the GenBank nonredundant database using the basic local alignment search tool (BLAST 2.0) [23] from the National Center for Biotechnology Information (http://www.ncbi.nlm.nih.gov/blast/) and were compared to the *R. prolixus *genomic database.

### Real-time quantitative polymerase chain reaction (qPCR)

The reactions were conducted using an ABIPRISM 7500 Sequence Detection System (Applied Biosystems). Each reaction was run in triplicate and contained 2 μl of diluted cDNA (corresponding to 0.1 ng of the starting amount of RNA for *18S *rRNA and 10 ng for the other genes), 300 nM of each primer and 12.5 μl of Power SYBR^® ^Green PCR Master Mix (Applied Biosystems) in a final volume of 25 μl. The cDNA was amplified at 95°C for 10 min followed by 40 cycles of 95°C for 15 s and 60°C for 1 min. A reverse transcription negative control (without reverse transcriptase) and a non template negative control were included for each primer set to confirm the absence of genomic DNA and to check for primer-dimer or contamination in the reactions, respectively. To ensure that only a single product was amplified, a melting curve analysis was performed. The real-time PCR efficiency was determined for each gene using the slope of a linear regression model [24], which was determined by measuring the C_T _for a specific threshold for a range of serial dilutions, including 100, 10, 1, 0.1 and 0.01 ng of cDNA. The corresponding real-time PCR efficiencies were calculated according to Radonic et al. [14].

### Statistical analysis

Raw C_T _values were first analyzed by the Kolmogorov-Smirnov test to check for normality. The non-parametric Mann-Whitney test was applied using the GraphPad Prism^® ^5.0 considering probabilities of P < 0.01 as significant [25]. Quantitative expression measurements of all of the candidate genes tested across different tissues and conditions obtained by qPCR were analyzed by the three statistical algorithms most commonly used for assessing the appropriateness of reference genes: geNorm [13], NormFinder [26] and BestKeeper [27], which are all freely available for download from the authors' websites.

## Results

### Target amplification efficiencies and expression levels of the candidate reference genes

The initial screening of five potential reference genes by PCR showed that all of the genes were expressed in *R. prolixus *salivary glands and crop, indicated by the presence of a single amplicon of the expected size on a 2% agarose gel. All of the amplicons were sequenced and displayed > 98% identity with the sequences from which the primer design was based. The efficiencies of the qPCR reactions were calculated from the formula E = 10^1/-slope ^- 1 and varied from 90.1% for *TUB *to 100% for *18S*; the regression coefficients ranged from 0.9617 to 0.9991 for *18S *and *L26*, respectively (Table 1).

**Figure 1:**
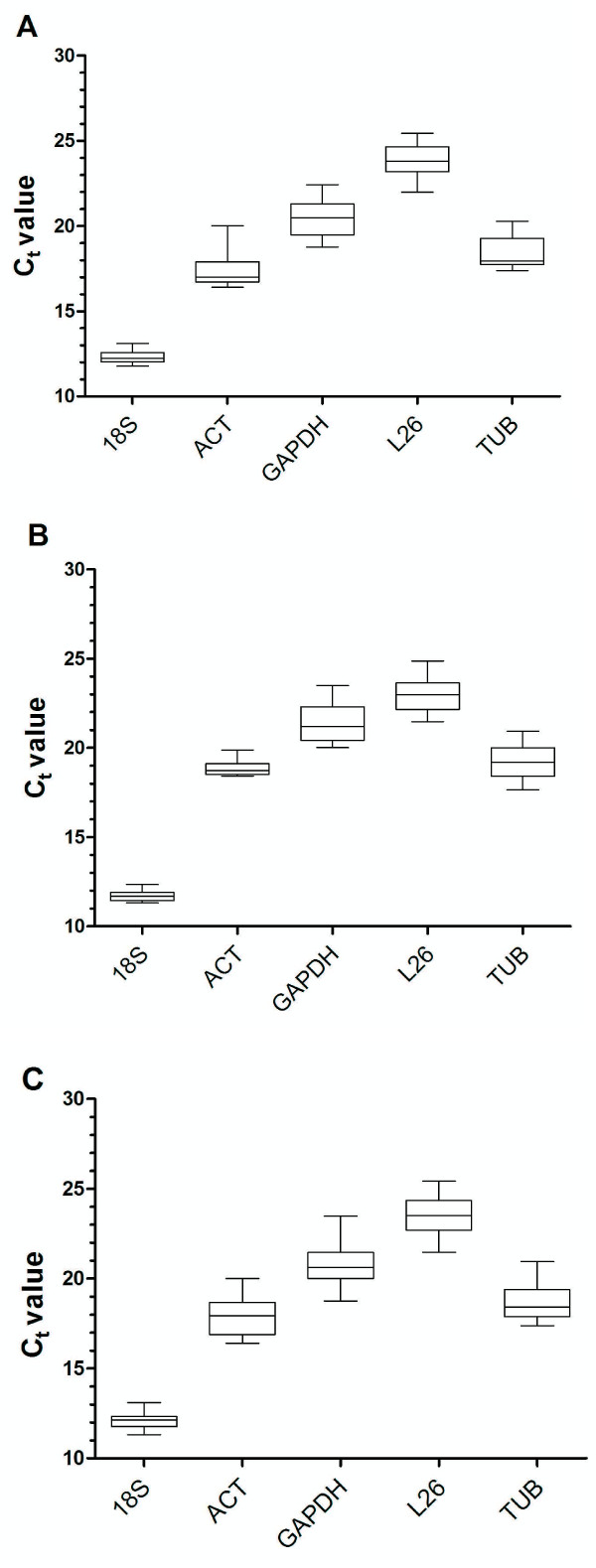
**The variation in gene expression in the tissues as indicated by the raw C_T _values**. The box plots show the expression levels of the candidate reference genes in (A) all of the crop samples (n = 22), (B) all of the salivary glands samples (n = 15) and (C) all of the tissues and conditions (n = 37). The values are given as the cycle threshold (C_T_, mean of triplicate samples). The global expression levels of the different genes analyzed are shown as the 25th and 75th quartiles (horizontal lines), median (emphasized horizontal line) and minimal to maximal value (whiskers)

The analysis of the raw expression levels identified variation among the reference genes (Figure 1). The C_T _values for the mRNAs selected as the candidate reference genes (Additional file 1) ranged from 11.31 (*18S*) to 25.42 (*L26*), and these two transcripts showed the most and the least abundant expression levels, respectively, in both tissue types tested (Figure 1a and 1b). The average C_T _values obtained for all of the genes studied did not show significant differences between the crop and the salivary gland, but the range of values was consistently narrower in the crop than in the salivary glands, except for the *ACT *gene (Figure 1a and 1b). qPCR amplification of *18S*, which is generally highly expressed in triatomine cells, produced C_T _values much lower than the other transcripts in all of the samples (C_T _mean = 12.07) despite the additional 1:100 dilution of the cDNA template. The other candidate reference genes were expressed at moderate levels, with mean C_T _(n = 37 samples) values of 17.93, 18.69, 23.49, and 20.82, respectively, for *ACT*, *TUB*, *L26 *and *GAPDH *(Figure 1c).

### Gene expression stability of the candidate reference genes

Ct values obtained in each situation showed that the variation of the gene expression was not significant (p > 0.01) in any organ and treatment (Table 2). Therefore, all of them were used for the analysis to identify the best reference genes.

**Table 2:** P values calculated by the two tailed Mann-Whitney test with the raw C_T _values of the genes in the salivary gland and crop after bloodfeeding* or infection** with *T.cruzi *or *T. rangeli*

	Salivary Gland		Crop		
	
	Blood feeding	*T. rangeli *infection	Blood feeding	*T. rangeli *infection	*T. cruzi *infection
**GAPDH**	1.000	0.036	0.057	0.071	1.000
**TUB**	0.857	0.036	0.629	0.051	0.686
**L26**	0.628	0.571	0.114	0.393	0.200
**ACT**	0.400	1.000	0.226	0.250	0.245
**18S**	0.400	0.714	0.226	0.571	0.886

* Comparison between before and after bloodfeeding

**Comparison between infected and non-infected

### geNorm analysis

**Figure 2:**
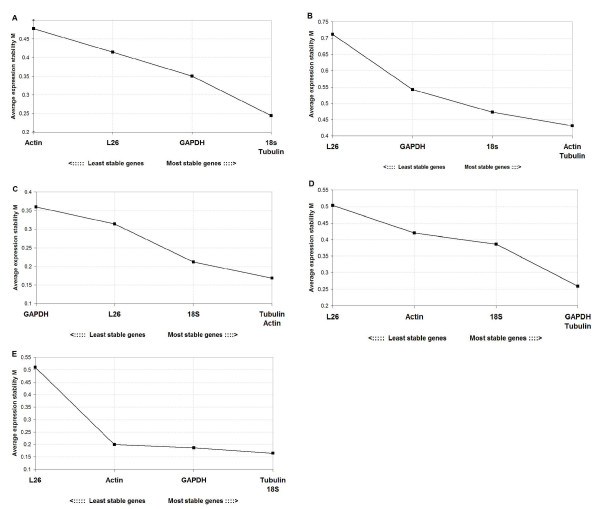
**Gene expression stability of the candidate reference genes using the geNorm software**. The average expression stability values (M) of the five reference genes were plotted from the least stable (left) to the most stable (right) in (A) the crop after blood feeding (n = 7 pools), (B) the salivary glands after blood feeding (n = 7 pools), (C) the crop after *T. rangeli *infection (n = 8 pools), (D) the salivary glands after *T. rangeli *infection (n = 8 pools) and (E) the crop after *T. cruzi *infection (n = 7 pools)

The geNorm program was used to identify genes that were the most stably expressed in different tissues or physiological conditions. The program defines two parameters to quantify the housekeeping gene stability: M (the average expression stability) and V (the pairwise variation). The gene with the lowest M value is considered to have the most stable expression, while the one with the highest M value has the least stable expression. All analyzed genes reached high expression stability with M-values below 0.5, except for L26 and GAPDH that showed M-values between 0.5 and 1.0 in three and one situation, respectively (Figure 2).

The gene that coded for α-tubulin proved to be one of the most stable genes in both tissues (salivary gland and crop) under the three experimental conditions (blood feeding, *T. rangeli *infection and *T. cruzi *infection). *18S *was also among the three most stable genes in all of the conditions. *ACT *expression was variable under blood feeding conditions, but showed highly stable expression in the salivary glands. However, *ACT *was the most unstable in the crop of the same insects. *L26 *was the least (or one of the least) stable genes in all of the tissues and evaluated conditions.

**Figure 3:**
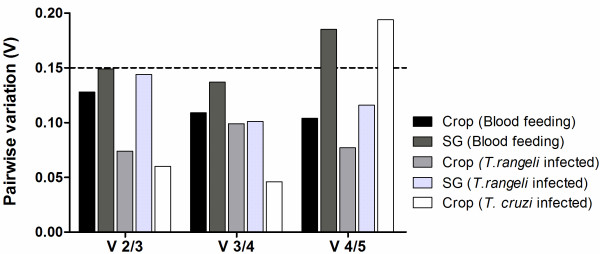
**The pairwise variation (V) of the candidate reference genes for accurate normalization in the two tissue samples in the different conditions: after blood feeding, *T. rangeli *infection or *T. cruzi *infection**. The threshold value (0.15) is marked with a dashed line and indicates that the use of only the two most stable genes was sufficient to obtain an accurate normalization in all of the analyzed groups (V 2/3 < 0.15). SG, salivary gland.

For all of the conditions (independent of the tissue or condition evaluated), combining the two most stably expressed genes produced optimal normalization. Indeed, inclusion of a third reference gene did not significantly contribute to the variation of the normalization factor because the V2/3 value (the pairwise variation when the number of normalization factors is increased from two to three) was below the default cut-off value of V = 0.15 [13] (Figure 3).

### BestKeeper analysis

According to the BestKeeper (Table 3) analysis, the candidate reference genes were stable in all of the tissues and experimental conditions because none showed SD values higher than 1. The single exception was the expression of *L26 *in the salivary glands after blood feeding, which presented the highest overall variation with an SD [± C_T_] > 1 and an SD [± x-fold] > 2, which makes its use as a reference gene unacceptable in that experimental condition. *18S*, *TUB *and *ACT *were among the three most stable genes in almost all of the conditions; these genes only varied in the rank position. Despite being at acceptable levels, *ACT *had the highest variation in the crop under blood feeding conditions. *L26 *and *GAPDH *exhibited the highest standard deviation in most of the conditions evaluated, indicating that these were the least stable reference genes.

**Table 3:** Descriptive statistics of the five candidate reference genes based on their cyclethreshold value (C_T_) as calculated by the BestKeeper algorithm

		*Ranking *→	*1*	*2*	*3*	*4*	*5*
***R. prolixus *crop**		**Gene name**	***18S***	***TUB***	***L26***	***GAPDH***	***ACT***

	Samples after blood feeding	Geo mean (C_T_)	12.66	19.45	24.38	21.68	18.28

		Min-max (C_T_)	12.25-13.10	19.18-20.27	23.51-24.86	21.23-22.43	18.88-20.0

		Std dev (± C_T_)	0.17	0.23	0.41	0.43	0.52

		Std dev (± x-fold)	1.12	1.18	1.33	1.35	1.43

	Samples after *T. rangeli *infection	Gene name	***ACT***	***TUB***	***18S***	***L26***	***GAPDH***

		Geo mean (C_T_)	16.93	17.70	12.06	23.73	20.53

		Min-max (C_T_)	16.64-17.11	17.38-18.14	11.78-12.48	23.1-24.67	19.88-21.24

		Std dev (± C_T_)	0.12	0.15	0.17	0.40	0.41

		Std dev (± x-fold)	1.09	1.11	1.12	1.32	1.33

	Samples after *T. cruzi *infection	Gene name	***18S***	***TUB***	***ACT***	***GAPDH***	***L26***

		Geo mean (C_T_)	12.21	18.02	16.66	19.22	23.33

		Min-max (C_T_)	11.91-12.44	17.86-18.35	16.39-17.30	18.76-19.72	21.99-25.42

		Std dev (± C_T_)	0.09	0.17	0.25	0.26	0.93

		Std dev (± x-fold)	1.07	1.13	1.19	1.20	1.90

***R. prolixus***							

**Salivary glands**	Samples after blood feeding	Gene name	***18S***	***ACT***	***TUB***	***GAPDH***	***L26***

		Geo mean (C_T_)	12.03	19.09	20.00	22.18	23.05

		Min-max (C_T_)	11.75-12.34	18.49-19.89	19.19-20.94	21.21-23.48	21.00-24.87

		Std dev (± C_T_)	0.23	0.46	0.47	0.65	1.17

		Std dev (± x-fold)	1.17	1.38	1.39	1.56	2.24

	Samples after *T. rangeli infection*	Gene name	***18S***	***ACT***	***GAPDH***	***TUB***	***L26***

		Geo mean (C_T_)	11.47	18.68	20.52	18.45	22.94

		Min-max (C_T_)	11.31-11.69	18.42-18.97	20.02-21.53	17.64-19.33	21.47-23.65

		Std dev (± C_T_)	0.11	0.19	0.26	0.39	0.49

		Std dev (± x-fold)	1.08	1.14	1.20	1.31	1.41

[C_T_], cycle threshold; Geo Mean [C_T_], geometric mean [C_T_]; Min-max [C_T_], extreme values of C_T_; Std dev [± C_T_], standard deviation [± C_T_]; Std dev [± *x*-fold], standard deviation of the absolute regulation coefficients

### NormFinder analysis

According to the NormFinder, the genes that are more stably expressed are indicated by lower average expression stability values. In contrast with the other two software analyses, the NormFinder algorithm identified *GAPDH *as one of the three most stable genes in almost all of the tissues and conditions, instead of *18S*, which often appeared in one of the top three positions in the other rankings (Table 4). An exception was the expression of *GAPDH *in the *R. prolixus *crop after *T. rangeli *infection in which *GAPDH *was the most unstable gene. *TUB *and *ACT *were among the three most stable genes in nearly all of the tissues and conditions, in agreement with the other two programs. The exception was the expression of *ACT *in the crop after blood feeding, which together with *L26*, was the least stable gene in this condition.

**Table 4:** Expression stability values from the candidate reference genes calculated by the NormFinder software

*R. prolixus s*alivary glands				*R. prolixus c*rop					
After blood feeding		After *T. rangeli *infection		After blood feeding		After *T. rangeli *infection		After *T. cruzi *infection	
**Ranking**	Stability value*	**Ranking**	**Stability value**	**Ranking**	**Stability value**	**Ranking**	**Stability value**	**Ranking**	**Stability value**
*ACT*	0.118	*GAPDH*	0.152	*TUB*	0.039	*TUB*	0.045	*ACT*	0.054
*GAPDH*	0.125	*TUB*	0.176	*GAPDH*	0.072	*ACT*	0.109	*GAPDH*	0.127
*TUB*	0.144	*ACT*	0.181	*18S*	0.078	*L26*	0.172	*TUB*	0.130
*18S*	0.185	*18S*	0.202	*L26*	0.168	*18S*	0.174	*18S*	0.188
*L26*	0.350	*L26*	0.224	*ACT*	0.168	*GAPDH*	0.215	*L26*	0.377
Best combination	Stability value	Best combination	Stability value	Best combination	Stability value	Best combination	Stability value	Best combination	Stability value
*GAPDH *and *ACT*	0.087	*TUB *and *ACT*	0.077	*GAPDH *and *TUB*	0.043	*TUB *and *ACT*	0.074	*GAPDH *and *ACT*	0.077

*The stability values are listed from the most stable to the least stable gene

The NormFinder algorithm also ranked the various candidate reference genes according to their intragroup expression variation. The comparisons were performed with organs and treatments that had subgroups, such as the salivary glands and crop with different times during starvation (subgroups: 1, 3, 15 and 25 days of starvation) and with different times after blood feeding (subgroups: 1, 3 and 7 days after feeding) (Table 5). In the salivary glands, *L26 *was the most variable gene (with the highest stability value) in both starving and feeding groups, while in the crop, it was only the most variable gene in starving insects. When the crop was compared at time-points after blood feeding, the most variable gene was *ACT*. This result reinforces the unacceptable use of *ACT *as a reference gene in this condition.

**Table 5:** Intra-group variation represented by the stability values calculated by the NormFinder software in the salivary glands and the crop of starving and blood-fed insects.

Tissue	Salivary Glands		Crop	
**Group identifier**	**Starving**	**Feeding**	**Starving**	**Feeding**
*GAPDH*	0.010	0.121	0.000	0.060
*TUB*	0.014	0.156	0.002	0.010
*L26*	0.413	0.428	0.143	0.064
*ACT*	0.019	0.084	0.000	0.333
*18S*	0.206	0.062	0.057	0.004

Intra-group variation close to zero indicates high stability

## Discussion

qPCR has become the most important method for the quantification of mRNA transcription levels due to its outstanding accuracy, broad dynamic range, and sensitivity. Normalization is a very important preliminary phase in the study of gene expression and requires the selection of a reference control, the expression of which may be influenced by experimental treatments in different tissues, which could lead to a misinterpretation of the results [28]. In triatomines, a variety of housekeeping genes has been frequently used in gene expression analysis. Among them, the most commonly used are *18S *rRNA [5,6,29,30] and β-actin [31-35]. Other genes, including the gene that codes for the protein UGALT, have also been suggested as housekeeping genes [11]. Although *UGALT *has been shown to be stable in groups with similar physiological status [36], its mRNA levels varied in nymphs at different days after feeding (data not shown). Recently, Majerowicz et al. evaluated seven genes in different organs of *R. prolixus *and identified that the best reference gene varied according to the tissue and physiological condition. Elongation factor 1 was the best in the posterior midgut, β-actin in the ovaries, 18 s ribosomal RNA in the fat body and embrionic lethal, abnormal vision in the posterior midgut infected with *T. cruzi *[18].

*Rhodnius prolixus *is currently being studied in several scientific projects, and the number of studies on *R. prolixus *is increasing due to the completion of the sequencing of its genome and the availability of an increasing number of transcribed sequences. Much attention is been given to organs related to the hematophagic process (such as the intestine and salivary glands), once they are important for the interaction between the vector and the host and are involved in the development and transmission of pathogens. The genes evaluated in the present study were selected based on their traditional use as internal controls in expression studies with triatomines (*ACT *and *18S*) and other organisms (*GAPDH*, ribosomal proteins and tubulin) [17], and also by their availability in the *R. prolixus *Genbank. We also attempted to test other reference genes that are widely used in several organisms, such as *HPRT *(hypoxanthine-guanine phosphoribosyltransferase), ubiquitin and *TBP *(TATA-box binding protein), but their sequences are not currently available in the *R. prolixus *databases.

The five candidate reference genes chosen were analyzed by three different statistical models because we assumed that a comparison of different algorithms enables a better evaluation of the most reliable reference genes and avoids the selection of co-regulated transcripts [37]. The sofwares geNorm, BestKeeper and NormFinder are the most common statistical algorithms used for reference gene validation, are easy to use and freely available for download. The geNorm software [13] is one of the most commonly used algorithms to compare the expression stability levels among different transcripts. The authors suggest that at least two reference genes should always be used to ensure accurate normalization and that each candidate reference gene should be validated before its use to ensure that it is stably expressed under the given experimental conditions. The geNorm analysis identified a pair of genes as the best reference genes for use in *R. prolixus *studies. In addition, the program can determine the optimal number of genes required for accurate normalization by calculating pair-wise variations. The major problem of the geNorm algorithm is its sensitivity to co-regulation, which tends to select those genes with the highest degree of similarity in their expression profiles [26].

The BestKeeper algorithm estimates the variability in the expression of the candidate reference genes by calculating the C_T _data variations and by performing a comparative analysis based on pairwise correlations of all of the candidate gene combinations. The gene expression variation was calculated for the five candidate genes, and the standard deviation (SD) provided an estimate of their stability.

NormFinder is another Excel-based visual basic application that assigns stability values to single candidate reference genes. Similar to geNorm, the NormFinder algorithm is based on expression values rather than the C_T_, uses a model-based approach for the estimation of expression variation among the candidate genes and takes into account variations among and within subgroups. Low stability values indicate less variation in gene expression. It also avoids misinterpretations caused by the artificial selection of co-regulated genes. The advantage of NormFinder in comparison to the two other programs is its ability to analyze the candidate reference genes according to their intra- and inter-group expression variations.

The results obtained from the three programs used in this study indicated that the stability of all five of the tested reference genes were under the threshold value required for a transcript to be considered a reliable reference gene. The exceptions were the *L26 *gene in the salivary glands in the blood feeding condition and *ACT *in the crop of blood-fed insects, which were considered unacceptable by the BestKeeper and Normfinder analysis, respectively. Moreover, L26 and GAPDH were identified by the geNorm with M-values above 0.5, which is the cut-off used by Hellemans et al. [38] to classify as stably expressed genes in relatively homogeneous samples. In nearly all of the conditions, the three programs classified *L26 *among the two least stable genes and GAPDH was the gene that showed more ranking variation among programs. Although their use as reference gene was considered acceptable, it should be used with caution because there are other more suitable reference genes. Previous works with triatomines [18] and mosquitoes [39] have shown that the expression of GAPDH has a transcription regulation profile in intestinal tissues after the bloodmeal.

The housekeeping gene data sets from the geNorm and BestKeeper analyses were similar, with slight differences in the first positions of the ranking order. Instead of *18S*, which often appeared as one of the three most stable genes in the geNorm and BestKeeper analyses, the NormFinder algorithm identified *GAPDH *as one of the three most stable reference genes. The best combinations of two genes proposed by the geNorm and NormFinder algorithms were different. Variation among programs was previously observed [40,41] and is mainly due to the different statistical algorithms used by each of them. GeNorm uses as most important criteria the expression ratio of the two genes evaluated while NormFinder is based in the least intra and inter group variations in gene expression and BestKeeper considers the stability (SD) and relationship to the BestKeeper index (r and p values) calculated by the software.

Our results showed that *18S *demonstrated high expression stability for almost all of the samples analyzed by the geNorm and BestKeeper algorithms, and this finding enables its use as a housekeeping gene. *18S *rRNA was previously shown suitable to be used as reference gene in other *R. prolixus *organs such as the posterior midgut, and fat body [18]. However, the use of rRNA as an endogenous control for qPCR has been criticized because it does not always represent the mRNA fraction [42] and the high abundance of rRNA makes it difficult to accurately subtract the baseline value for the qPCR data analysis. Jiang et al. [43] reported the inappropriate use of the *18S *rRNA gene as a reference in studies with the insect *Liposcelis bostrychophila*. The rRNA constitutes the largest fraction of RNA in the cell (up to approximately 80%), while the mRNA corresponds to approximately 5% of the total RNA. Thus, normalization errors can be caused by variations in cellular rRNA, which will change the total RNA content [44].

*TUB *was also among the top of the rankings of the most stable genes in all of the conditions regardless of the program used. Consequently, it can be used as a reference gene for general use in qPCR normalization in hematophagy and triatomine- trypanosomatid interaction studies.

The results obtained in this work confirmed that *ACT*, one of the most widely used genes, is a suitable and reliable reference gene for triatomine studies to compare different treatments in the same organ. This gene displayed high stability in the salivary glands and the crop in almost all of the experimental conditions. However, in the crop of insects subjected to blood feeding, *ACT *was the worst housekeeping gene with the lowest expression stability. This finding was observed in all three of the software programs and indicates that the use of *ACT *as a reference gene should be avoided in this experimental condition. Moreover, Majerovicz et al. showed that *ACT *expression levels vary considerably between tissues and it should not be used to compare different organs [18]. Similar results were reported in studies of cellular growth and differentiation [45]. During and after hematophagy of *R. prolixus *(as for other triatomines), the crop (the portion of the intestine that stores the ingested blood) volume significantly increases with the blood input to the digestive tract, and the abdominal cuticle needs to stretch to support the diet [46]. β-actin plays key roles in cell motility and cytoskeleton maintenance [47], and its expression in intestinal cells is probably affected by the morphological alterations caused by blood feeding.

## Conclusions

In conclusion, this work defines the appropriateness of candidate genes for use as qPCR reference genes in the salivary glands and crop of *R. prolixus *studies. Our data showed that the expression stability of the genes varies in different tissue samples and under different experimental conditions. The results provided by three easily accessible statistical packages emphasize the suitability of all five of the tested reference genes in both the crop and the salivary glands with only a few exceptions. The gene (or combination of genes) to be used as a reference should be chosen depending on the experimental conditions. Under the conditions used here, the geNorm analysis suggested that the combined use of the two most stable genes is sufficient for an accurate normalization in all of the tissues and conditions analyzed.

## Abbreviations

cDNA: Complementary DNA; PCR: Polymerase chain reaction; qPCR: Quantitative real-time polymerase chain reaction; *TUB*: α-tubulin; Ct: Cycle threshold; ACT: β-actin; GADPH: Glyceraldehyde-3-phosphate- dehydrogenase; SD: Standard deviation; *UGALT*: UDP-galactose translocator; Tm: Melting temperature

## Competing interests

The authors declare that they have no competing interests.

## Authors' contributions

RMP performed all the experimental procedures, data analysis, write the manuscript and was the primary author of the manuscript. MHP, NFG and AAG assisted in manuscript revising and provided helpful discussions. RDP performed salivary glands and intestine dissections and sample preparations. JOR maintained the trypanosomatids culture and infected the triatomines. RNA conceived and supervised the research. All authors read and approved the final manuscript.

## Supplementary Material

Additional file 1 **Table of single Ct values of all candidate reference genes evaluated in this study in salivary glands and in the intestine in each experimental condition**. Ct mean and standard deviation of Ct values of candidate reference genes. Click here for file
