# Supplementary material for: Validation of reference genes for expression analysis in the salivary gland and the intestine of Rhodnius prolixus (Hemiptera, Reduviidae) under different experimental conditions by quantitative real-time PCR
Source: BMC Res Notes. 2012 Mar 6;5:128. doi: 10.1186/1756-0500-5-128 (PMC3337225; doi:10.1186/1756-0500-5-128)
Supplement: Additional file 1 — Table of single Ct values of all candidate reference genes evaluated in this study in salivary glands and in the intestine in each experimental condition. Ct mean and standard deviation of Ct values of candidate reference genes. [file 1756-0500-5-128-S1.PDF]

Feeding status- starving and fed

	Ct- Intestine					Ct- Salivary glands				
Sample	**18S**	**ACT**	**GAPDH**	**L26**	**TUB**	**18S**	**ACT**	**GAPDH**	**L26**	**TUB**
1	12.25	18.04	21.41	24.72	19.30	12.34	18.62	22.39	22.15	20.29
2	12.64	17.89	21.29	24.42	19.46	12.24	19.89	22.85	24.83	20.94
3	12.54	17.88	21.23	23.51	19.18	11.91	18.55	21.39	21.90	19.59
4	12.82	17.94	21.31	23.80	19.29	11.90	18.49	21.71	22.18	19.19
5	12.66	17.90	22.29	24.62	19.26	12.31	19.60	23.48	24.87	20.45
6	12.65	18.38	21.85	24.75	19.43	11.75	19.11	21.21	22.03	20.00
7	13.10	20.00	22.43	24.86	20.27	11.79	19.41	22.31	23.63	19.63
Mean Ct	12.67	18.29	21.69	24.38	19.46	12.03	19.10	22.19	23.08	20.01
SD	0.25	0.77	0.50	0.52	0.37	0.25	0.55	0.81	1.33	0.59

Infection status- *T. cruzi* infected and non-infected

Ct- Intestine

Sample	**GAPDH**	**TUB**	**L26**	**ACT**	**18S**
1	19.54	18.31	23.67	16.82	12.26
2	19.17	17.94	24.21	16.75	12.21
3	19.72	18.35	25.42	17.30	12.44
4	18.76	17.89	21.99	16.49	11.91
5	19.31	17.92	22.87	16.43	12.23
6	18.96	17.86	22.62	16.49	12.24
7	19.09	17.91	22.68	16.39	12.19
8	19.74	19.12	21.84	16.94	12.65
Mean Ct	19.29	18.16	23.16	16.70	12.27
SD	0.36	0.43	1.21	0.31	0.21

Infection status- *T. rangeli* infected and non-infected

	Ct- Intestine					Ct- Salivary glands				
Sample	**GAPDH**	**TUB**	**L26**	**ACT**	**18S**	**GAPDH**	**TUB**	**L26**	**ACT**	**18S**
1	19.88	17.66	23.76	16.86	12.24	20.55	18.74	22.69	18.43	11.69
2	19.98	17.38	23.36	16.64	12.06	20.64	18.85	23.65	18.59	11.64
3	20.34	17.53	23.22	16.94	11.96	21.57	19.33	23.41	18.90	11.46
4	20.32	17.66	23.10	16.82	11.91	20.42	18.45	23.24	18.97	11.31
5	20.83	17.82	23.87	16.91	12.14	20.45	18.17	23.44	18.52	11.34
6	20.61	17.75	24.25	17.07	11.95	20.33	18.03	22.70	18.73	11.44
7	21.24	18.14	24.67	17.07	12.48	20.02	17.64	21.47	18.88	11.34
8	21.10	17.68	23.66	17.11	11.78	20.35	18.31	22.97	18.42	11.52
Mean Ct	20.54	17.70	23.74	16.93	12.06	20.54	18.44	22.95	18.68	11.47
SD	0.50	0.22	0.53	0.16	0.22	0.45	0.53	0.69	0.22	0.14
